# Online training and support program (iSupport) for informal dementia caregivers: protocol for an intervention study in Portugal

**DOI:** 10.1186/s12877-019-1364-z

**Published:** 2020-01-08

**Authors:** Soraia Teles, Ana Ferreira, Katrin Seeher, Stéfanie Fréel, Constança Paúl

**Affiliations:** 1 Department of Behavioral Sciences, Institute of Biomedical Sciences Abel Salazar, University of Porto (ICBAS-UP), Rua de Jorge Viterbo Ferreira, 228, 4050-313 Porto, Portugal; 2 Center for Health Technology and Services Research (CINTESIS), Rua Dr. Plácido da Costa, 4200-450 Porto, Portugal; 3 Faculty of Medicine, University of Porto (FMUP), Alameda Prof. Hernâni Monteiro, 4200-319 Porto, Portugal; 4 Department of Mental Health and Substance Use (MSD), World Health Organization, Avenue Appia 20, 1202 Geneva, Switzerland

**Keywords:** Informal caregivers (IC), People with dementia (PwD), Online training and support, Intervention study

## Abstract

**Background:**

Informal caregivers (IC) of people with dementia (PwD) are at greater risk of developing physical and mental health problems when compared to the general population and to IC of people with other chronic diseases. Internet-based interventions have been explored for their potential to minimize the negative effects of caring, accounting for their ubiquitous nature, convenient delivery, potential scalability and presumed (cost) effectiveness. iSupport is a self-help online program developed by the World Health Organization (WHO) to provide education, skills training and support to IC of PwD. This paper describes the design of an intervention study aimed at determining the effectiveness of a Portuguese culturally adapted version of iSupport on mental health and other well-being outcomes.

**Methods:**

The study follows an experimental parallel between-group design with two arms: access to the five modules and twenty-three lessons of “iSupport” for three months (intervention group); or access to an education-only e-book (comparison group). One hundred and eighty four participants will be recruited by referral from national associations. Inclusion criteria are: being 18 years or older and provide e-consent; being a self-reported non-paid caregiver for at least six months; of a person with a formal diagnosis of dementia; being skilled to use internet; and experience a clinically relevant level of burden (≥ 21 on Zarit Burden Interview) or depression or anxiety symptoms (≥ 8 on Hospital Anxiety and Depression Scale). Data is collected online, resorting to self-administered instruments, at baseline, 3 and 6 months after baseline. The primary outcome is caregiver burden, measured by the Zarit Burden Interview. Symptoms of depression and anxiety, quality of life, positive aspects of caregiving and general self-efficacy are secondary study outcomes. The data analysis will follow an Intention-to-treat (ITT) protocol.

**Discussion:**

This protocol is an important resource for the many organizations in several countries aiming to replicate iSupport. Findings from this intervention study will offer evidence to bolster an informed decision making on scaling up iSupport as a new intervention program with minimal costs aimed at minimizing the psychological distress of IC of PwD in Portugal and elsewhere.

**Trial registration:**

ClinicalTrials.gov, NCT04104568. Registered 26 September 2019.

## Background

Worldwide, there are 9.9 million new cases of dementia each year, 2.5 million of those identified in Europe [1]. In 2015, it was estimated that 47.5 million people lived with dementia across the globe [2]. Dementia is a syndrome caused by neurodegenerative diseases affecting memory, thinking, behaviour and the ability to perform daily activities. Among older adults, this group of diseases is one of the main causes of disability and dependence, thus people with dementia (PwD) often require long term or permanent care [3].

Caring responsibilities are mostly assumed by family members and other informal caregivers, especially in lower-middle income countries and in countries characterized by a familialistic tradition [4–6]. Informal caregivers (IC) provide unpaid and ongoing assistance with basic or instrumental activities of daily living to a person with a disability or chronic illness [7]. While those caregivers may experience positive intrinsic reinforcement from the experience of caring, they are also often exposed to several stressors including financial problems, time constraints and care management issues [8]. As a consequence, when compared with both the general population and IC of people with other chronic diseases, IC of PwD are at a greater risk of developing depression and anxiety disorders, as well as hypertension, digestive, and breathing problems [8–11]. Caregivers’ psychosocial variables - including the experience of stress, burden and mental disorders, and the perceived inability to provide care - were previously related with abusive or harmful behaviours towards the care recipients [12], and identified as predictors of the care receiver’s institutionalization [13–15]. Reducing the negative effects of caring is instrumental to prevent undesired upshots for both the IC and the care receivers.

As higher levels of stress tend to be experienced by a person if coping resources are perceived to be less than one demands, IC tend to benefit from looking for external resources that might support them in dealing with stress, as well as in providing PwD with quality care [16, 17]. Interventions targeting IC vary in their style of administration and content, including psychoeducational interventions, supportive interventions, respite care, psychotherapy, interventions to improve care receivers’ competence/skills, and multicomponent interventions [18]. Regarding the efficacy or effectiveness of those interventions, some outcomes may be more responsive to certain types of interventions than to others and effect sizes may be influenced by the intervention type (specific effects) [18]. Current evidence suggests more robust effects for psychoeducational and multicomponent interventions (including, e.g. skills training, psychoeducation, techniques for self-care, changes in the caregiver’s setting) on subjective well-being, caregiver burden, depression and anxiety symptoms, skills/knowledge and self-efficacy [10, 18–21].

In spite of demonstrated beneficial effects of such interventions on numerous caregiver outcomes, several reports have been stressing the existence of situational barriers impeding IC of PwD of accessing it when delivered in its usual face-to-face format [8, 22–25]. Barriers may hamper the offer of those intervention programs (e.g. geographical inequalities, lack of trained workforce, infrastructures to scale up services, and/or funds); or may prevent IC from participating (e.g. social stigma, not managing to make a break from caregiving responsibilities or to arrange transport) [8].

To overcome such barriers, online training and support programs have been increasingly explored, accounting for their ubiquitous nature, convenient and privacy-preserving delivery, as well as potential scalability and presumed (cost)effectiveness [25–27]. The number of scientific publications on the use, acceptability and effectiveness of Information and Communication Technologies (ICT)-based interventions for IC of PwD (including but not limited to internet-based interventions) increased by 13% each year from 1990 to 2016 [28]. Several recent systematic reviews and meta-analysis examined the evidence on the effectiveness of internet-based intervention programs delivered to IC of PwD on a plethora of outcomes [29–33]. Beneficial effects were mostly reported on the reduction of anxiety and depression symptoms [29–33], perceived stress and burden [31–33] (not found in [29]), as well as on the increase of self-efficacy [31, 32], even if effects can be small [31]. Multicomponent interventions, tailored to individual needs, offering contact with other caregivers and coaching were reported as most beneficial [31, 33]. Reviews not defining specific outcomes as inclusion criteria for intervention studies show that most frequently studied outcomes include depression and anxiety symptoms and overall mental health, stress and burden, self-efficacy, knowledge, use of health services, positive aspects of caregiving and quality of life [31, 33]. Instruments used to measure such outcomes vary widely. In spite of some evidence in favor of internet-based interventions, inconsistent interventions (e.g. varied structure, duration, outcomes) and a substantial methodological diversity across studies tend to compromise a pooling of results [30, 31]. Moreover, the methodological quality of studies vary greatly and the lack of research details and quality reports seem to be important problems in the available literature [29]. In some cases, the overall evidence was considered low quality, hampering the interpretation and generalizability of the findings on effectiveness [31, 33]. Selection, performance, and reporting biases were present and a reason for concern [30, 31, 33], hence more well-thought research is welcome in this field.

Recently, grounded in the evidence-based guidelines and recommendations of the World Health Organization's (WHO) Mental Health Gap Action Programme (mhGAP) [34], WHO convened a panel of international experts to develop “iSupport”, a self-help online training and support program designed to provide education, skills training and social support to IC of PwD. iSupport was released in its generic version to be adapted to specific countries and populations. Owing to identified needs, iSupport was translated to European-Portuguese and culturally adapted to the Portuguese context. The country presents a high share of old (+ 65 years) and very old (+ 80 years) people (21.5 and 6.3% respectively, in 2018) [35]; is positioned above the OECD average (Organisation for Economic Co-operation and Development) and ranks fourth in terms of dementia prevalence, with an estimation of 19.9 cases of dementia per 1000 inhabitants in 2017 [36]; and has one of the highest rates of informal home care in Europe (12.4%) [37].

The effectiveness of culturally adapted vs. non-adapted interventions on clinical outcomes has been compared by several systematic reviews and meta-analysis and yielded mixed evidence [38–46]. Effect sizes ranged from almost zero (e.g. [44]) to large effect sizes favoring culturally adapted interventions (e.g. [40, 45]). Regarding iSupport, two intervention studies with culturally adapted versions of the program are ongoing: a first study, concluded but not published yet, carried out in a lower-middle income country, in India, using an English but culturally adapted version of the program [47]; and a second, currently recruiting participants, carried out in the Netherlands (unpublished observations; Pinto et al., 2019). The extent to which the Portuguese culturally adapted version of iSupport is effective on mental health and other well-being outcomes is unknown and must be determined. This paper describes the design of an intervention study to be carried out in Portugal to determine the effectiveness of a Portuguese culturally adapted version of an online training and support program for IC of PwD (iSupport), on specified mental health and other well-being outcomes.

## Methods

### Type of study, aims and research question

The study follows an experimental parallel between-group design with two arms aiming to determine the effectiveness of an online training and support program (iSupport, European-Portuguese version), as compared to a minimal education-only intervention (e-book on dementia and caregiving issues), to decrease caregivers’ burden, symptoms of depression and anxiety, and to increase quality of life, positive aspects of caregiving and self-efficacy, in IC of PwD. The intervention study is set in Portugal and planned to last six months. The research question - Can the Portuguese version of iSupport reduce perceived burden (primary outcome) and improve mental health, quality of life, and coping skills (secondary outcomes), in informal caregivers of people with dementia? - is addressed by the intervention study discussed in this paper. The Additional file 1 provides the SPIRIT checklist for recommended items to address in a protocol, applied to this intervention study.

### Selection of participants

#### Target population, inclusion and exclusion criteria

Informal caregivers of people with dementia are screened to assess whether they are (i) Portuguese adults (≥18 years); (ii) giving consent to participate (Electronic Informed Consent); (iii) providing non-paid care for at least 6 months at the time of the recruitment; (iv) caring for a person holding a formal diagnosis of dementia; and (v) be skilled to use the internet. For inclusion, the IC (vi) must also experience either a clinically relevant level of subjective burden, determined by a total score ≥ 21 on the Zarit Burden Interview (ZBI) [48, 49] or depression or anxiety symptoms, determined by a score ≥ 8 in at least one of the subscales of the Hospital Anxiety and Depression Scale (HADS) [50, 51]. Participants matching the inclusion criteria will be excluded if (i) they are unable to comprehend written Portuguese or (ii) do not have access to a device with internet connection at least twice a week. If (iii) the care receiver is in institutional care (e.g. nursing home or continued care unit), the participant is also excluded. How each inclusion and exclusion criteria are ascertained is described below (see 2.2.2. Sampling and recruitment). Both excluded participants and participants assigned to the control group will be offered the opportunity to use iSupport after the study closure, in case of manifested interest.

#### Sampling and recruitment

**Fig. 1:**
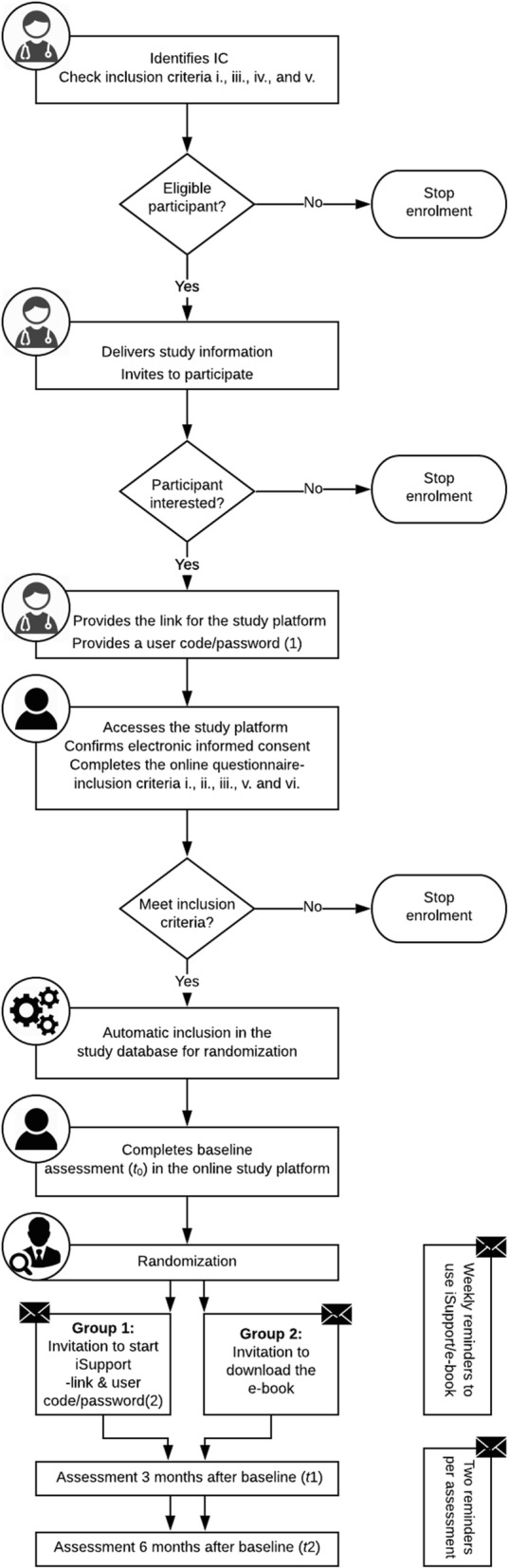
Participant selection flowchart

A consecutive (nonprobability) sampling strategy is employed, by consecutively selecting subjects who meet the entry criteria. Potential participants are referred by health professionals from national Alzheimer’s associations (not for profit organizations offering support in the community to IC of PwD) who are aware of the formal dementia diagnosis of the care receivers. Due to feasibility constraints, an independent diagnosis or second diagnosis of the care receivers will not be obtained. The process of recruiting and selecting the participants is illustrated in Fig. 1. First, health professionals identify the IC of PwD and verify the compliance with inclusion criteria i., iii., iv. and v. (see section Target population, inclusion and exclusion criteria). IC complying with these criteria and not matching the exclusion criteria are provided with the participant information sheet and invited to express their interest in enrolling in the study. IC that have expressed their interest are provided with a link to the online study platform and a user code/password (1) to access the platform. In case the participants declining to participate express their motives for taking that decision, those motives are noted with their consent, as a mean to control for barriers to participation and selection bias. Upon accessing the study online platform for the first time (at the time and place of their convenience), participants must provide an email contact and authorize its use to receive information about the study and will be prompted to complete a questionnaire aimed at determining or double-checking the compliance with inclusion criteria i., ii., iii., v., and vi. (see section Target population, inclusion and exclusion criteria). While the referral process aims to minimize the chances of volunteer bias, matching a specific referred person with a user accessing the platform would require complex arrangements, thus inclusion criteria verified at the referral process are double-checked with the online questionnaire. Due to feasibility constraints, a clinical evaluation of burden, anxiety and depression symptoms (inclusion criteria vi.) will not be conducted, instead these symptoms will be assessed by well-established self-report instruments (see sectionVariables and data collection methods) delivered via the study online platform. If the potential participants have a score indicating severe symptoms of anxiety and/or depression, a message is displayed in the screen recommending the participant to consult local mental health services.

**Table 1:**
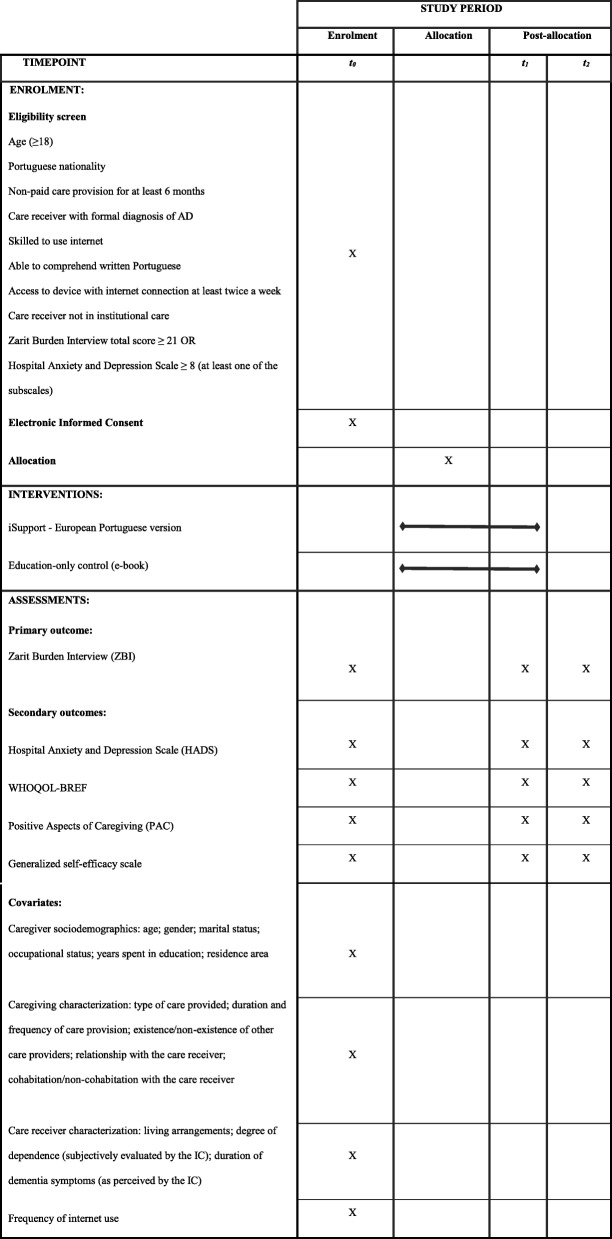
Schedule of enrolment, interventions, and assessments for iSupport intervention study.

Eligible participants will be automatically included in a database to be randomized and assigned to either the intervention or control arm. Included participants will be prompted on the screen to continue filling in the questionnaire in order to complete the baseline assessment (*t*_0_). This means that a single questionnaire is programmed into the study platform and organized in a hierarchical and conditional manner: for participants not meeting the inclusion criteria, the system will terminate the questionnaire while for included participants, the system will display all study instruments for the baseline assessment. Of note some questionnaire items are used both to verify inclusion criteria and to measure covariates (e.g. caregiving duration) or outcomes (caregiver burden; symptoms of depression and anxiety). With the list of participants consecutively included in the database, the researcher applies the randomization key to allocate the IC to the intervention or control group (see section Randomization and blinding). In accordance to the allocated group, the participant receives, by email, either a user code/password (2) to access the iSupport platform or a link to download the e-book.

### Randomization and blinding

Eligible participants are randomly assigned to the intervention or to the control arm by using a permuted block randomization strategy to maintain reasonably good balance among groups. Blocks of four with equal number of As (intervention) and Bs (control) will be used, with the order of interventions within the block being randomly permuted (1 = AABB, 2 = ABAB, 3 = ABBA, 4 = BAAB, 5 = BABA, 6 = BBAA). Baseline characteristics of individuals randomized to each condition will be compared to verify if the randomization was successful. Health professionals referring the participants to the study platform are not aware of the randomization sequence, and even if they were, they could not control the participants’ allocation as they could not predict the sequence status at the time of the participants’ access to the study platform. Similarly, the researcher assigning the participants to each arm has no control over the order of enrolment (i.e. over who will access the online platform and when), and differs from the researcher performing the statistical analysis at a subsequent stage. The data analyst will be blinded to the intervention assigned to the participants. The study is single-blinded: participants are not unaware of the intervention received as they are active parts of the intervention. Data collection at baseline and outcome assessments are carried out in a computerized manner via study platform and resorting to self-administered instruments, thus the researcher has no influence over the measurements.

### Variables and data collection methods

Data collection will be carried out at three different time points: i. at baseline (*t*_0_); ii. post-intervention (3 months after baseline; *t*_1_); and iii. follow-up (6 months after baseline; *t*_2_). Table 1 presents the schedule of interventions and assessments. Primary and secondary outcomes are measured through self-report instruments composing the data collection protocol. All data will be collected in a computerized manner through the study online platform. Three and six months after the baseline (*t*_1_ and *t*_2_), the participants are invited by email to fill in the online questionnaires and, as an attempt to avoid missing data, two reminders are foreseen for each assessment (see Fig. 1).

#### Intervention vs. control condition

The independent variable/factor is a dichotomous variable describing the intervention with iSupport or a control with a minimal education-only intervention (e-book).

##### iSupport for dementia – European-Portuguese version

**Fig. 2:**
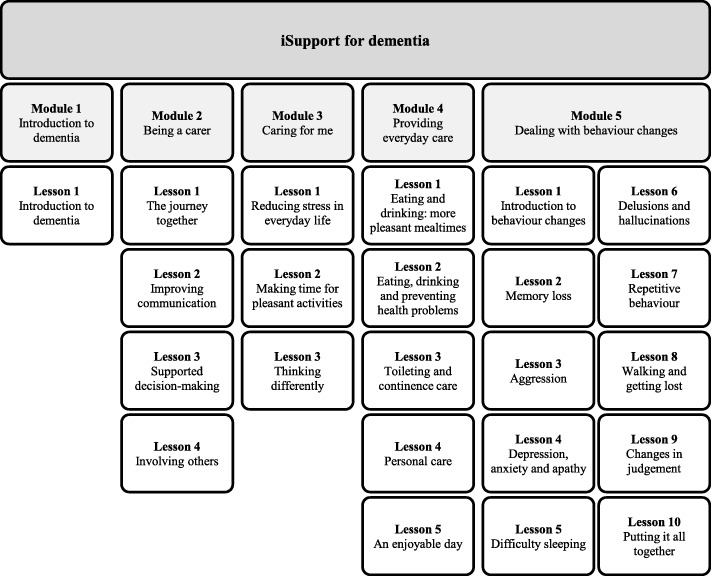
Overview of the iSupport online self-help training and support program structure and contents. Adapted from iSupport Adaptation and Implementation Guide (unpublished observations; Pot, 2018). For illustrative purposes the English version of module and lesson titles is presented

The intervention group will be provided with access, for 3 months, to an online self-help training and support program: the iSupport for dementia – European-Portuguese version. The e-program offers information, skills training and support for IC of PwD. It comprises five modules, including twenty-three lessons covering well-established topics on dementia and caregiver support. Figure 2 provides an overview of the program structure, summarizing the thematic contents addressed. In line with good practices on digital engagement [52], the education plan can be personalized by the caregiver. This means that it can be adjusted to the person’s availability and lessons can be selected according to particular needs. Each lesson includes interactive exercises with immediate feedback; and positive messages as well as ‘skills certificates’ are displayed when lessons are completed aiming at increasing adherence. From a methodological perspective, those elements are not understood as prompting a differential follow up bias for both arms - the comparison condition does not offer feedback and positive messages - as those are part of the intervention features. Framed as a multi-component intervention, iSupport is grounded in problem-solving and cognitive behavioural therapy techniques including psycho-education, behavioural activation, cognitive reframing, relaxation and antecedent-behaviour-consequence (ABC) analysis (unpublished observations; Pot, 2018).

The process of culturally adapting the program to the European Portuguese language and Portuguese culture is described elsewhere (unpublished observations; Teles, Napolskij, Paúl, Ferreira and Seeher, 2019). In brief, the Portuguese version of iSupport was adapted following a five step procedure including needs assessment; content translation by authorized translator and technical accuracy check by health professionals; cultural adaptation; independent appraisal of contents by an expert panel; and fidelity check by program authors. Moreover, end-user feedback is accounted for by conducting focus groups and a field-testing/piloting along with a usability study. For the Portuguese version, adjustments to the original contents were needed, concerning semantic and conceptual equivalence of expressions, and adjustments to cultural habits, customs, traditions, local resources, knowledge and practices. The fidelity check by WHO ensured that adaptations were in line with the core concepts of the original program, while tailoring iSupport to the Portuguese context was critical to accommodate the cultural experiences of the target-group as well as the knowledge, theoretical approaches, and practices of local professionals.

Together with access to iSupport, the participants allocated to the intervention group will receive weekly reminders to use the program (see Fig. 1).

##### Comparison condition: education-only

The control group will receive a minimal education-only intervention, consisting on an e-book created by the European Commission and Alzheimer Europe [53], and translated to European-Portuguese by the Association Alzheimer Portugal [54]. The manual contains information on relevant topics for dementia and caregiving, including basic information about dementia; practical tips on managing dementia; the changing needs of a person with dementia; dealing with care provision and with the death of the care receiver; caring for oneself and finding emotional support; relevant legislation (e.g. advance directives); and how to get help and reach the national Alzheimer’s Association. In Portugal there is no standard care in what concerns support to IC of PwD; besides sporadic face-to-face training and support initiatives, the most common support given to IC of PwD is in the form of informative material (e.g. leaflets, books, online resources) provided by health and social work professionals. The e-book selected is among the most complete resources for IC of PwD available in Portugal. Together with access to the e-book, the participants allocated to the control group will receive weekly reminders to read the manual, as a measure to avoid bias due to different follow-up (as reminders are provided to the intervention group).

#### Outcomes

The outcomes of this study have been widely used in intervention studies assessing the effectiveness of internet-based support tools [27]. The primary outcome is caregiver burden measured with the Zarit Burden Interview (ZBI), Portuguese validated version [48, 49], total score, at *t*_1_. The instrument comprises 22 items answered on a 5-point scale, ranging from 0 (never) to 4 (almost always) except for the final question on global burden, rated from 0 (not at all) to 4 (extremely). Total score for the ZBI ranges from 0 to 88 points, with higher scores indicating greater burden. The ZBI has good internal consistency (e.g. α = 0.89 in [55]), test-retest and interrater reliability as well as construct and concurrent validity, including reports of positive correlation with depression symptoms and time devoted to care provision [56, 57]. Regarding the psychometric properties of ZBI in Portugal, face, content, ecological, discriminant and convergent validity were documented, along with test-retest reliability (ICC = 0.93, CI95% 0.88–0.96, *p* < 0.001) and internal consistency (*α* = 0.88) [57]. The ZBI is one of the most used instruments to measure burden in intervention studies with caregivers [18].

The following variables are included as secondary outcomes: (i) symptoms of depression and anxiety measured through the Portuguese version of the Hospital Anxiety and Depression Scale (HADS) [50, 51], total scores. The instrument comprises two subscales with 7 items each, using a 4-point scale. Total scores for each scale range from 0 to 21, with higher scores indicating more severe anxiety or depression symptoms; (ii) quality of life measured by the Portuguese version of the WHOQOL-BREF physical, psychological, social relationships and environment [58, 59], total transformed scores. The instrument contains 26 items, for total transformed scores ranging between 0 to 100 points, with higher scores denoting higher quality of life; (iii) positive role appraisals mediating between the stressor and caregiver well-being, measured through the Portuguese version of Positive Aspects of Caregiving (PAC) [57, 60], total score. This instrument comprises 11 items answered in a 5-point scale, for a total score ranging between 11 to 55 points and higher scores representing more positive appraisals; and (iv) general self-efficacy, measured through the total score of the Generalized Self-efficacy Scale [61, 62]. The scale comprises 10 items answered in a 4-point scale for a total score ranging between 10 to 40 points, and higher scores representing a higher general self-efficacy. The instruments used for data collection can be found in the references provided or under request to the authors of each instrument. The variables defined as primary and secondary outcomes are assessed at baseline and repeated measurements are performed at *t*_1_ and *t*_2_. An attempt to reach participants from both groups who do not complete the assessments will be made in order to collect the reasons.

#### Covariates

Variables measured at baseline (*t*_0_) include sociodemographic characteristics of IC, namely their age, gender, marital and occupational status, years spent in education and residence area. In addition, a questionnaire for caregiving characterization collects information on the type of care provided, i.e. paid/non-paid (screening); duration (number of years providing care) and frequency of care provision (number of hours per week); existence/non-existence of other care providers; relationship with the care receiver; and cohabitation/non-cohabitation with the care receiver. Moreover, age and gender of the care receiver are collected, as well as information on the care receiver’s living arrangements (living in the community vs. institutional care; screening), on the degree of care receiver’s dependence (as subjectively evaluated by the IC); and on the duration of dementia symptoms (as perceived by the IC). ICT usage is characterized by the frequency of internet use.

### Sample size calculation

As a baseline for estimating the sample size, the primary null hypothesis was stated:

*H0: The change in mean caregiver burden from baseline at t*_1_ *is the same in informal caregivers of people with dementia assigned to the iSupport program as in those assigned to a minimal education-only intervention.*

A two-sided alternative hypothesis was assumed:

*H1: The change in mean caregiver burden from baseline at t*_1_ *is different in informal caregivers of people with dementia assigned to the iSupport program as in those assigned to a minimal education-only intervention.*

Caregiver burden at *t*_1_ (measured with the ZBI) is the primary outcome measure, therefore used for the sample size calculation. The effect size was defined taking into account a meta-analysis reporting a mean standardized effect size of 0.65 (95% confidence interval [0.46–0.84]), for multicomponent interventions, on caregiver burden [18]. Assuming a smaller but still moderate standardized effect size of 0.5 (E/S), equivalent to about a 6-point endpoint mean difference, and a SD of 12 based on Bachner and Rourke [63] and assuming an *α* (two-sided) = 0.05 and a β = 0.2, a sample size of 64 per group was calculated as follows:

*Standardized effect size (E/S) = 6 ÷ 12 = 0.5.*

16 ÷ (standardized effect size)^2^ = 16 ÷ 0.25 = 64

Conservatively allowing for a larger dropout rate of 30% found in the literature for these interventions [47], a total of 92 informal caregivers per group was calculated:

16 ÷ 0.25 × 1.43 = 92

With a sample size of 92 per group and a dropout rate no larger than 30%, the study will be able to detect an intervention effect of 0.5 for the primary outcome.

### Statistical analysis

Descriptive statistics will be used to characterize the IC enrolled in the intervention study: means and standard deviations for the continuous variables presenting normal distribution; medians and percentiles for continuous variables not normally distributed or ordinal variables; frequencies for nominal variables. At *t*_0_, the randomization success will be examined by comparing the characteristics of participants in both arms with the adequate statistical methods. Since the outcomes are continuous variables the best measure is the change in the outcome over the course of the study: this approach usually minimizes the outcome variability between participants and offers more power than just comparing values at the end of the study. To evaluate differences between the outcomes of each group, paired t-tests will be conducted and estimates of effect sizes calculated. All *p* values are two-sided with a significance level of 0.05. Multiple regression analyses can be used to correct for possible confounders. Generalized estimated equations (GEE) analysis can be used to examine the maintenance of the intervention effectiveness. The data will be analyzed by a researcher blinded to the group assignment.

The data analysis will follow an Intention-to-treat (ITT) protocol, therefore participants will be analyzed as randomized. This approach is an important protection against bias by preserving the randomization benefits, in particular by balancing known and unknown factors and eliminating selection bias. As the ITT approach requires either no attrition or a strategy to handle missing data, to minimize the first, reminders for both assessments (*t*_1_ and *t*_2_) are foreseen and to address the second, missing data on follow-up measurements will be imputed using multiple imputations. In addition, a per protocol sensitivity analysis will be conducted to ‘bracket’ the likely effects under different conditions. For this purpose, a minimum number of completed lessons (*n* = 5), in addition to the completion of the assessments (*t*_1_ and *t*_2_), will be considered. An interim analysis is foreseen, to be implemented before the participants’ enrolment is completed. As the intervention study does not raise relevant concerns about intervention safety, a single look/interim assessment, when half of the sample is reached, is planned. Elements to monitor in the interim analysis include recruitment, adherence to intervention, follow-up completeness, and reported harms. Measures that might arise from the interim analysis include extending the study in time, increasing the study sample or stopping the study. The study should be stopped in case of determination of benefit or harm, or in case of being identified a negligible chance of demonstrating effectiveness if the intended number of participants were fully enrolled. Only the research team will have access to the interim results and make the final decision to terminate the trial if fulfilled the stopping criteria. To minimize type I error, the *α* will be decreased in the interim analysis. All analyses will be conducted using SPSS for Windows.

## Discussion

The development of accessible, acceptable and effective training and support interventions for IC of PwD is a strategic priority in the global action plan on the public health response to dementia 2017–2025 [64]. By considering the promising benefits of internet-based intervention programs, as well as its accessibility, potential for scalability and residual delivery costs, iSupport might be a valuable resource to be used in dementia care. In the Portuguese context, a scalable approach to relieve caregivers’ psychological distress is much needed, given the high dementia prevalence and high rate of informal home care (see Background).

In this paper, we outlined the design of an intervention study to determine the effectiveness of a Portuguese, culturally adapted version of iSupport, to decrease caregiver burden (primary outcome) and symptoms of depression and anxiety as well as to increase quality of life, positive aspects of caregiving and self-efficacy (secondary outcomes), as compared to a minimal education-only intervention (e-book), in IC of PwD. Choosing a comparison condition for this study was not straightforward as no standard care is available for dementia caregivers in Portugal. Even if intervention programs are in place, there is no single one used at national level. The hardcopy manual of iSupport could be used as a comparator; however, by doing so, the underlying assumption is that only the mode of delivery (paper vs. online) would be compared rather than the contents of the program per se. In the absence of a standard care, the most broadcasted resource on caregiving so far in Portugal (an e-book) was selected as the comparator.

The outcomes defined for this study, as well as the instruments selected to measure them, have been widely used when researching the effectiveness of intervention programs, internet-based or not, aimed at supporting caregivers. This is the case for caregiver burden and the classic ZBI instrument. However, the definition of caregiver burden, measured through ZBI, as the primary outcome, has some disadvantages of which the researchers are fully aware of. First, certain outcomes may be more responsive to specific types of interventions than to others: interventions with a considerable focus on increasing caregivers’ ability and knowledge are more prone to promote changes on those dimensions than on burden, depression or anxiety symptoms and other well-being outcomes [18]. Even though iSupport is framed as a multicomponent intervention, the provision of information and skills training are strong components of this program. Second, some outcome measures may be more sensitive to changes than others, this being the case for caregiver burden, usually less modifiable than, for instance, measures of knowledge or ability. Indeed, some studies report no significant effects of interventions on burden or record smaller effects on this outcome than on the previous [18]. Lower effect sizes on burden might also be found when burden is measured with the ZBI [18]. As stressed by Zarit and Leitsch, a lower sensitiveness of burden measures to change is not surprising, as even the most successful interventions can reduce the impact of the care provision but they are unable to eliminate the problem [65]. Altogether, these aspects are demanding in terms of sample size, so the study can have the power to detect smaller intervention effects. Therefore, a successful recruitment of participants is a key aspect of this study and encompasses most of its risks. Recruitment by referral from health professionals (by opposing to a recruitment by advertising) was chosen for this intervention study with the purpose of minimizing the likelihood of volunteer bias and misleading self-reports. However, this strategy also carries more study risks as it is more dependent on successful collaborations with health professionals and dementia organizations.

The enrolment of participants in this study may be limited by some noteworthy aspects. First, while the daily use of the internet in the Portuguese population has grown substantially – this has doubled in less than 10 years - and 79% of Portuguese homes had access to internet in 2018 [66], it should not be ignored that the country is still positioned below the EU-28 average with regards to internet use by all individuals (64% vs. 76%) [67]. Second, iSupport would be an unique tool in the Portuguese context, i.e. no other internet-based intervention with these features is available in Portugal for IC of PwD, which may limit its initial acceptance. Moreover, among IC of PwD, the overall use of internet-based resources to gather information and support on dementia and caregiving issues seems to be moderate to low, as previously concluded by the authors from a web-based survey (unpublished observations; Teles, Ferreira and Paúl, 2019). Indeed, while internet use by IC of PwD for health and caregiving-related purposes varied greatly across specific purposes (e.g. gather information of the disorders; learn about strategies to provide quality care; obtain support for the caregivers), it was never observed in more than 50% of the sample (unpublished observations; Teles, Ferreira and Paúl, 2019). However, a more frequent use of internet for these purposes was reported by IC classifying the care receivers’ degree of dependence as ‘total’ or ‘severe’; or assessing their own physical health as ‘much worse’ or ‘worse’ when compared to their counterparts (unpublished observations; Teles, Ferreira and Paúl, 2019). This might suggest that caregivers with pressing support needs could be more open to use internet-based resources. In view of anticipated challenges for recruiting participants, this study can always be used to assess the feasibility of an internet-based intervention program for Portuguese IC of PWD.

Regarding the intended sample for this study, it should be noted that there are limitations to the generalization of the intervention effects from that sample, directly represented by the trial, to the entire population of IC of PwD. Eligible IC for this study must be digitally literate and have access to a device with internet connection, conditions that are likely to favor the enrolment of younger and highly educated participants (as education level is a well-known determinant of internet usage [68]), not representing the mainstream population of IC of PwD. Also, participants are more likely to live in urban settings, where better infrastructures for internet provision are available. Still, to our understanding, those IC represent the target group of internet-based intervention tools, which naturally tend to reach a segment of (digitally literate) caregivers.

Important protocol modifications are not foreseen. However, imperative unforeseen protocol changes will be dully communicated to relevant parties if and when they arise. There might be a possibility for this study to resort to qualitative methods and data collection techniques to answer a broader range of evaluation questions of a processual nature. While the counterfactual analysis from an intervention study demonstrates *if* and *to what extent* the intervention works, a factual analysis based on qualitative data allows to address the *how* and *why* the intervention works/does not work. In the scope of assessing iSupport, exploring the quality of implementation, barriers to participation or adoption, and unmeasured perceived benefits, might be particularly useful due to: the unfeasibility of measuring all interesting outcomes documented in the literature that might be affected by this kind of intervention; low sensitiveness of some outcome measures to change; and due to the expected obstacles in participants recruitment and high dropout rates. A selection of participants might be invited to be interviewed at the end of the study to explore the abovementioned aspects.

This protocol is an important resource for the many organizations in several countries aiming to replicate iSupport. Ultimately, evidence from several iSupport country-specific versions will provide insights on the program’s effectiveness. It will also enhance current knowledge regarding the extent to which culturally adapted evidence-based interventions retain their core properties and effectively fulfill their inteded purpose. This intervention study should provide valuable insights for practitioners, researchers and policy makers regarding the feasibility and effectiveness of internet-based resources for supporting IC of PwD, thus improving informed decision making on the use of such resources. When the study is completed, the Portuguese version of iSupport will be made available free of charge. If promising results are shown, iSupport would be an unique program in the Portuguese context, given that the program fully aligns with the national strategic action plans on dementia, ageing and mental health [69].

## Supplementary information

**Additional file 1.** SPIRIT 2013 Checklist for recommended items to address in a protocol

**Additional file 2.** Participant information form and consent form

## Data Availability

Not applicable.

## Abbreviations

ABC: Antecedent-behaviour-consequence analysis
E/S: Standardized effect size
EU: European Union
GEE: Generalized estimated equations
HADS: Hospital Anxiety and Depression Scale
IC: Informal caregivers
ICT: Information and Communication Technologies
ITT: Intention-to-treat
mhGAP: WHO’s Mental Health Gap Action Programme
OECD: Organisation for Economic Co-operation and Development
PAC: Positive Aspects of Caregiving
PwD: People with Dementia
SD: Standard deviation
SPSS: Statistical Package for the Social Sciences
T0: Baseline measurement
T1: Post intervention measurement
T2: Follow-up measurement
WHO: World Health Organization
ZBI: Zarit Burden Interview
