# Supplementary material for: Online training and support program (iSupport) for informal dementia caregivers: protocol for an intervention study in Portugal
Source: BMC Geriatr. 2020 Jan 8;20:10. doi: 10.1186/s12877-019-1364-z (PMC6950829; doi:10.1186/s12877-019-1364-z)
Supplement: Supplementary file 1 — Additional file 1. SPIRIT 2013 Checklist for recommended items to address in a protocol [file 12877_2019_1364_MOESM1_ESM.doc]

**Additional file 1** - SPIRIT 2013 Checklist for recommended items to address in a protocol.

**
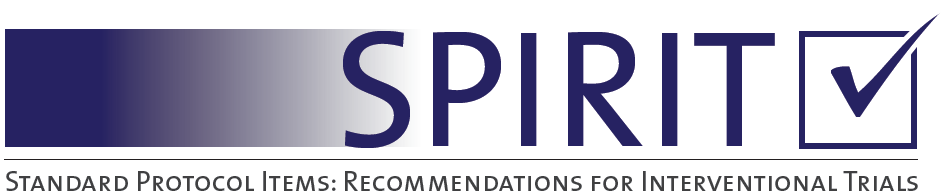
**

| Section/item | Item No | Description | Addressed on section and page number |
| --- | --- | --- | --- |
| **Administrative information** | | |  |
| Title | 1 | Descriptive title identifying the study design, population, interventions, and, if applicable, trial acronym | p. 1 |
| Trial registration | 2a | Trial identifier and registry name. If not yet registered, name of intended registry | ClinicalTrials.gov, ID: NCT04104568 |
|  | 2b | All items from the World Health Organization Trial Registration Data Set (https://www.who.int/ictrp/network/trds/en/) | See section “Ethics approval and consent to participate” for items 1-3, 5-6, 10-11, 21, 24 (p. 15); see section “Funding” for item 4 (p. 16); section “Authors’ information and contacts” for items 7-8 (p. 17); title for item 9 (p. 1); section “Outcomes” for items 12 and 19-20 (p. 9); section “Intervention vs. control condition” for item 13 (p. 7); section “Target population, inclusion and exclusion criteria” for item 14 (p. 5); section “methods” for item 15 (p. 5); see “trial status” for item 16 and 18 (p. 15); section “Sample size calculation” for item 17; n/a for item 22 (p. 10). See registry at ClinicalTrials.gov, ID: NCT04104568. |
| Protocol version | 3 | Date and version identifier | See section “Trial status” (p. 15) |
| Funding | 4 | Sources and types of financial, material, and other support | See section “Funding” (p. 16) |
| Roles and responsibilities | 5a | Names, affiliations, and roles of protocol contributors | See section “Authors’ contributions” (p. 16) |
|  | 5b | Name and contact information for the trial sponsor | See Authors’ information and contacts (p. 17) |
|  | 5c | Role of study sponsor and funders, if any, in study design; collection, management, analysis, and interpretation of data; writing of the report; and the decision to submit the report for publication, including whether they will have ultimate authority over any of these activities | See section “Funding” (p. 16) |
|  | 5d | Composition, roles, and responsibilities of the coordinating centre, steering committee, endpoint adjudication committee, data management team, and other individuals or groups overseeing the trial, if applicable (see Item 21a for data monitoring committee) | NA |
| Introduction |  |  |  |
| Background and rationale | 6a | Description of research question and justification for undertaking the trial, including summary of relevant studies (published and unpublished) examining benefits and harms for each intervention | See sections “Background” and “Type of Study, aims and research question” (p. 3, 5) |
|  | 6b | Explanation for choice of comparators | See section “Intervention vs. control condition” (p. 7) |
| Objectives | 7 | Specific objectives or hypotheses | See sections “Type of Study, aims and research question” and “Sample size calculation” (p. 5) |
| Trial design | 8 | Description of trial design including type of trial (eg, parallel group, crossover, factorial, single group), allocation ratio, and framework (eg, superiority, equivalence, noninferiority, exploratory) | See section “methods” sub-sections “Type of Study, aims and research question” (p. 5), “Randomization and blinding” (p. 7) and “Sample size calculation” (p. 10) |
| Methods: Participants, interventions, and outcomes | | |  |
| Study setting | 9 | Description of study settings (eg, community clinic, academic hospital) and list of countries where data will be collected. Reference to where list of study sites can be obtained | See sections “Type of Study, aims and research question” (p. 5) and “Sampling and recruitment” (p. 6) |
| Eligibility criteria | 10 | Inclusion and exclusion criteria for participants. If applicable, eligibility criteria for study centres and individuals who will perform the interventions (eg, surgeons, psychotherapists) | See section “Target population, inclusion and exclusion criteria” (p. 5) |
| Interventions | 11a | Interventions for each group with sufficient detail to allow replication, including how and when they will be administered | See section “Intervention vs. control condition” (p. 7) |
|  | 11b | Criteria for discontinuing or modifying allocated interventions for a given trial participant (eg, drug dose change in response to harms, participant request, or improving/worsening disease) | NA |
|  | 11c | Strategies to improve adherence to intervention protocols, and any procedures for monitoring adherence (eg, drug tablet return, laboratory tests) | See section “Intervention vs. control condition” (p. 7) |
|  | 11d | Relevant concomitant care and interventions that are permitted or prohibited during the trial | NA |
| Outcomes | 12 | Primary, secondary, and other outcomes, including the specific measurement variable (eg, systolic blood pressure), analysis metric (eg, change from baseline, final value, time to event), method of aggregation (eg, median, proportion), and time point for each outcome. Explanation of the clinical relevance of chosen efficacy and harm outcomes is strongly recommended | See sections “Outcomes” (p. 9) and “Statistical analysis” (p. 11) |
| Participant timeline | 13 | Time schedule of enrolment, interventions (including any run-ins and washouts), assessments, and visits for participants. A schematic diagram is highly recommended (see Figure) | See table 1 Schedule of enrolment, interventions, and assessments for iSupport intervention study (p. 10). |
| Sample size | 14 | Estimated number of participants needed to achieve study objectives and how it was determined, including clinical and statistical assumptions supporting any sample size calculations | See section “sample size calculation” (p. 5) |
| Recruitment | 15 | Strategies for achieving adequate participant enrolment to reach target sample size | See section “Sampling and recruitment” (p. 6) |
| **Methods: Assignment of interventions (for controlled trials)** | | |  |
| Allocation: |  |  |  |
| Sequence generation | 16a | Method of generating the allocation sequence (eg, computer-generated random numbers), and list of any factors for stratification. To reduce predictability of a random sequence, details of any planned restriction (eg, blocking) should be provided in a separate document that is unavailable to those who enrol participants or assign interventions | See section “Randomization and blinding” (p. 7) |
| Allocation concealment mechanism | 16b | Mechanism of implementing the allocation sequence (eg, central telephone; sequentially numbered, opaque, sealed envelopes), describing any steps to conceal the sequence until interventions are assigned | See sections “Sampling and recruitment” (p. 6) and “Randomization and blinding” (p. 7) |
| Implementation | 16c | Who will generate the allocation sequence, who will enrol participants, and who will assign participants to interventions | See sections “Randomization and blinding” (p. 7) and “Sampling and recruitment” (p. 6) |
| Blinding (masking) | 17a | Who will be blinded after assignment to interventions (eg, trial participants, care providers, outcome assessors, data analysts), and how | See section “Randomization and blinding” (p. 7) |
|  | 17b | If blinded, circumstances under which unblinding is permissible, and procedure for revealing a participant’s allocated intervention during the trial | NA |
| **Methods: Data collection, management, and analysis** | | |  |
| Data collection methods | 18a | Plans for assessment and collection of outcome, baseline, and other trial data, including any related processes to promote data quality (eg, duplicate measurements, training of assessors) and a description of study instruments (eg, questionnaires, laboratory tests) along with their reliability and validity, if known. Reference to where data collection forms can be found, if not in the protocol | See section “Variables and data collection methods” (p. 7) |
|  | 18b | Plans to promote participant retention and complete follow-up, including list of any outcome data to be collected for participants who discontinue or deviate from intervention protocols | See section “Variables and data collection methods” (p. 7) |
| Data management | 19 | Plans for data entry, coding, security, and storage, including any related processes to promote data quality (eg, double data entry; range checks for data values). Reference to where details of data management procedures can be found, if not in the protocol | See section “Ethics approval and consent to participate” (p. 15) for data security and storage; no comments on plans for data entry, coding or other processes to promote data quality. |
| Statistical methods | 20a | Statistical methods for analysing primary and secondary outcomes. Reference to where other details of the statistical analysis plan can be found, if not in the protocol | See section “Statistical analysis” (p. 11) |
|  | 20b | Methods for any additional analyses (eg, subgroup and adjusted analyses) | See section “Statistical analysis” (p. 11) |
|  | 20c | Definition of analysis population relating to protocol non-adherence (eg, as randomised analysis), and any statistical methods to handle missing data (eg, multiple imputation) | See section “Statistical analysis” (p. 11) |
| **Methods: Monitoring** | | |  |
| Data monitoring | 21a | Composition of data monitoring committee (DMC); summary of its role and reporting structure; statement of whether it is independent from the sponsor and competing interests; and reference to where further details about its charter can be found, if not in the protocol. Alternatively, an explanation of why a DMC is not needed | NA |
|  | 21b | Description of any interim analyses and stopping guidelines, including who will have access to these interim results and make the final decision to terminate the trial | See section “Statistical analysis” (p. 11) |
| Harms | 22 | Plans for collecting, assessing, reporting, and managing solicited and spontaneously reported adverse events and other unintended effects of trial interventions or trial conduct | See section “Ethics approval and consent to participate” (p. 15) |
| Auditing | 23 | Frequency and procedures for auditing trial conduct, if any, and whether the process will be independent from investigators and the sponsor | NA |
| Ethics and dissemination | | |  |
| Research ethics approval | 24 | Plans for seeking research ethics committee/institutional review board (REC/IRB) approval | Approved, see section “Ethics approval and consent to participate” (p. 15) |
| Protocol amendments | 25 | Plans for communicating important protocol modifications (eg, changes to eligibility criteria, outcomes, analyses) to relevant parties (eg, investigators, REC/IRBs, trial participants, trial registries, journals, regulators) | See section “Ethics approval and consent to participate” (p. 15) |
| Consent or assent | 26a | Who will obtain informed consent or assent from potential trial participants or authorised surrogates, and how (see Item 32) | See sections “Target population, inclusion and exclusion criteria” (p. 5) and “Ethics approval and consent to participate” (p. 15) |
|  | 26b | Additional consent provisions for collection and use of participant data and biological specimens in ancillary studies, if applicable | NA |
| Confidentiality | 27 | How personal information about potential and enrolled participants will be collected, shared, and maintained in order to protect confidentiality before, during, and after the trial | See section “Ethics approval and consent to participate” (p. 15) |
| Declaration of interests | 28 | Financial and other competing interests for principal investigators for the overall trial and each study site | See competing interests |
| Access to data | 29 | Statement of who will have access to the final trial dataset, and disclosure of contractual agreements that limit such access for investigators | See section “Ethics approval and consent to participate” (p. 15) |
| Ancillary and post-trial care | 30 | Provisions, if any, for ancillary and post-trial care, and for compensation to those who suffer harm from trial participation | NA |
| Dissemination policy | 31a | Plans for investigators and sponsor to communicate trial results to participants, healthcare professionals, the public, and other relevant groups (eg, via publication, reporting in results databases, or other data sharing arrangements), including any publication restrictions | See section “Dissemination policy” (p. 17) |
|  | 31b | Authorship eligibility guidelines and any intended use of professional writers | NA |
|  | 31c | Plans, if any, for granting public access to the full protocol, participant-level dataset, and statistical code | NA |
| Appendices |  |  |  |
| Informed consent materials | 32 | Model consent form and other related documentation given to participants and authorised surrogates | See Additional file 2 |
| Biological specimens | 33 | Plans for collection, laboratory evaluation, and storage of biological specimens for genetic or molecular analysis in the current trial and for future use in ancillary studies, if applicable | NA |
