# Supplementary material for: Online training and support program (iSupport) for informal dementia caregivers: protocol for an intervention study in Portugal
Source: BMC Geriatr. 2020 Jan 8;20:10. doi: 10.1186/s12877-019-1364-z (PMC6950829; doi:10.1186/s12877-019-1364-z)
Supplement: Supplementary file 2 — Additional file 2. Participant information form and consent form [file 12877_2019_1364_MOESM2_ESM.doc]

**Additional file 2** – Participant information form and consent form

INFORMAÇÃO AO PARTICIPANTE

**Estudo: Apoio e formação *online* para cuidadores informais de pessoas que vivem com demência: Estudo de intervenção.**

*Por favor, solicita-se que leia com atenção a informação que se segue. Se considerar que algo está incorreto ou que não está claro, não hesite em contactar o investigador principal do estudo no sentido de fazer os sus comentários ou solicitar mais informações. Após ponderar a sua participação e caso concorde com a proposta que lhe foi feita, queira, for favor, assinar o consentimento informado que lhe será fornecido.*

Caro(a) potencial participante,

É convidado a participar neste estudo porque foi referenciado como cuidador informal de uma pessoa que vive com demência. Devido aos desafios associados à prestação de cuidados a uma pessoa que vive com demência, sabe-se que os cuidadores informais destas pessoas têm maior probabilidade de experienciar sintomas de sobrecarga do cuidador assim como sintomas de ansiedade e depressão quando comparados com a população em geral.

A Organização Mundial de Saúde (OMS) desenvolveu um programa educativo e de apoio a cuidadores informais de pessoas que vivem com demência designado de “iSupport”. Este programa tem a particularidade de ser implementado online/através da internet, podendo o cuidador participar nas 23 sessões de intervenção que o compõem a qualquer momento e a partir do local que lhe seja mais conveniente, através dos seus dispositivos pessoais (computador, telemóvel, tablet). O iSupport foi identificado como um programa de intervenção promissor, tendo sido adaptado ao contexto Português mas não tendo sido ainda determinada a sua eficácia neste contexto. Assim, com vista à implementação alargada do programa no contexto Português é fundamental determinar a sua eficácia. O CINTESIS - Centro de Investigação em Tecnologias e Serviços de Saúde - encontra-se atualmente à procura de potenciais participantes interessados em contribuir para o estudo de intervenção do programa iSupport.

**Objetivos do estudo e metodologia a utilizar:** O objetivo desde estudo de intervenção é o de determinar a eficácia do programa iSupport (Organização Mundial de Saúde, 2017), particularmente quanto à redução da perceção de sobrecarga do cuidador assim como dos sintomas de ansiedade e depressão e quanto ao aumento da qualidade de vida, aspetos positivos do cuidar e autoeficácia geral, em cuidadores informais de pessoas que vivem com demência e por comparação a uma intervenção educativa mínima. Na prossecução deste objetivo recorre-se a um estudo de intervenção.

**Detalhes da participação:** Convidámo-lo(a) a fazer parte deste estudo de intervenção no qual os participantes - os cuidadores informais de pessoas que vivem com demência - poderão ser alocados a dois grupos distintos:

Grupo 1: Este grupo terá a oportunidade de realizar o programa iSupport educativo e de apoio *online*/através da internet, constituído por 23 sessões organizadas em 5 módulos. Estes participantes terão acesso ao programa, através de um nome de utilizador e palavra passe pessoais e intransmissíveis, durante 3 meses, podendo realizar as sessões individual e autonomamente no horário e local mais conveniente para si através dos seus dispositivos habituais (computador, tablet, telemóvel);

Grupo 2: Este grupo de cuidadores receberá, através do endereço de email, um livro digital educativo dedicado aos tópicos da demência e prestação de cuidados a pessoas que vivem com esta condição.

Os participantes serão alocados a cada um dos grupos aleatoriamente, como se fosse atirada uma moeda ao ar para decidir a que grupo cada participante pertencerá. Esta técnica é muito importante para garantir que ambos os grupos são comparáveis quanto a fatores conhecidos e desconhecidos o que é fundamental para assegurar que, caso um grupo tenha melhores resultados do que outro, tal se deve à intervenção que receberam e não a outros fatores.

Assim, caso aceite participar neste estudo, não saberá à partida a qual dos grupos irá pertencer, se ao grupo 1 ou ao grupo 2. No entanto, após o término do estudo, ser-lhe-á dada a possibilidade de aceder à intervenção que não realizou durante o estudo, se for essa a sua vontade.

A sua inclusão neste estudo depende do cumprimento de critérios de inclusão e do não cumprimento de critérios de exclusão:

1. Será elegível para incluir o estudo se: i. tiver mais de 18 anos; ii. der consentimento (através desta plataforma digital) para participar no estudo; iii. for um cuidador não remunerado há pelo menos 6 meses; iv. cuidar de alguém com diagnóstico formal de doença de Alzheimer; v. for capaz de aceder e utilizar a internet de forma independente e com alguma regularidade; e vi. experienciar sobrecarga do cuidador e sintomas de ansiedade ou depressão. Este último critério será avaliado com base nas suas respostas aos questionários iniciais.
2. Caso cumpra os critérios acima mas também se encontre nas seguintes situações, não será elegível para participar no estudo: i. se o seu recetor de cuidados vive numa instituição (por exemplo, num lar de terceira idade); ii. se não tem possibilidade de usar um dispositivo com acesso à internet pelo menos duas vezes por semana.

Tendo em conta estes critérios, pode acontecer que aceite participar neste estudo e não seja possível fazê-lo. Se for esse o caso, é-lhe dada a possibilidade de receber o livro digital educativo ainda que não seja considerado(a) enquanto participante do estudo.

Caso aceite participar no estudo ser-se-á pedido que responda, num primeiro momento, a um conjunto de questões acerca de: i. características socio-demográficas (por exemplo idade, sexo); ii. atividades de prestação de cuidados (por exemplo, quanto tempo dedica à prestação de cuidados, que relação tem com o recetor de cuidados); iii. uso de novas tecnologias (por exemplo, quantas vezes usa a internet); e iv. questões que permitem avaliar o seu nível de sobrecarga, sintomas de ansiedade e/ou depressão, aspetos positivos do cuidar, qualidade de vida e auto-eficácia. A resposta a estas questões poderá demorar até 1 hora, no máximo. Após responder a estas questões e caso preencha os critérios para participar, será então alocado ao grupo 1 ou ao grupo 2, recebendo ora as credenciais de acesso ao programa iSupport, ora o livro digital, através do seu endereço de email que deverá disponibilizar previamente. Uma vez por semana irá receber um lembrete que o recordará da utilização do programa ou do manual digital. Três e seis meses (2 momentos) após receber a informação correspondente ao grupo a que foi alocado(a) ser-lhe-á pedido que responda novamente a um conjunto de questões que permitem avaliar o seu nível de sobrecarga, sintomas de ansiedade e/ou depressão, aspetos positivos do cuidar, qualidade de vida e auto-eficácia. A resposta a estas questões poderá demorar aproximadamente 30 minutos. Todas as questões, colocadas em qualquer um dos três momentos acima descritos, serão respondidas através desta plataforma *online* do estudo.

**Condições da participação:**

A sua participação no estudo é totalmente voluntária, tendo total liberdade para decidir se pretende ou não participar. A qualquer momento pode ainda decidir desistir da participação no estudo sem que isso possa resultar em qualquer prejuízo para si ou comprometa o relacionamento com os profissionais que o referenciaram ou o respeito pelos direitos de assistência que lhe são devidos. Não se prevê o pagamento de contrapartidas associadas à sua participação. Após receber esta informação, ser-lhe-á dado tempo para refletir sobre o pedido de participação, inclusivamente para poder ouvir opinião de familiares e/ou amigos se assim o desejar. Prevê-se que esse tempo de reflexão seja de cerca de uma semana, com possibilidade de ajustamentos.

**Confidencialidade, anonimato e proteção de dados**: A informação que nos fornecer será anónima e confidencial. O termo de consentimento informado solicitar-lhe-á o nome e o número de BI/Cartão do Cidadão. Essa informação será tratada separadamente da informação fornecida com a resposta às questões acima descritas com vista a que o seu nome não seja associado à sua resposta às questões, quando analisadas. Todos os dados recolhidos neste estudo serão utilizados apenas pela equipa de investigação e para o propósito por si autorizado, não sendo nunca cedidos a qualquer outra instituição. Para o efeito de análise dos dados e conservação dos mesmos numa base de dados, ser-lhe-á atribuído um código apenas conhecido pela equipa de investigação e conservado em servidor seguro da Universidade do Porto, como de resto acontecerá com todos os ficheiros que contenham informação que possa identificá-lo (por exemplo, os consentimentos informados). Com efeito, a plataforma do estudo, onde poderá responder às questões mencionadas, está alocada nos recursos/servidores e moradas digitais da Universidade do Porto, logo numa plataforma segura que utiliza encriptação das comunicações para proteção dos participantes. Caso seja alocado ao grupo 1 (iSupport), os seus dados (registo e perfil de utilização) serão preservados nos servidores seguros da Organização Mundial de Saúde (OMS) sem que seja possível identifica-lo(a) através destes. Adicionalmente, os resultados deste estudo serão unicamente apresentados por meio de artigos e comunicações científicas nas quais, em momento algum, será identificado.

**Benefícios e riscos:** Não se anteveem riscos ou incómodos derivados da sua participação neste estudo. Uma vez que todos os procedimentos associados à participação e previamente descritos se desenvolvem *online*, não lhe será pedido que realize deslocações não habituais, tendo a possibilidade de aceder às sessões de intervenção e preencher os questionários no momento e local que lhe forem mais convenientes. Logo, a participação no estudo de intervenção não implica que se ausente do seu local de trabalho (com consequentes perdas de remuneração ou faltas associadas) ou que haja disrupção de qualquer outra atividade habitual. Adicionalmente, os dispositivos a serem usados para aceder ao programa/manual digital e responder aos questionários serão os seus dispositivos habituais sem que se preveja, assim, qualquer desconforto pela introdução de novos dispositivos.

Os benefícios associados à sua participação são ainda desconhecidos, sendo este estudo desenhado precisamente para os determinar. Estudos prévios com programas de intervenção online similares ao iSupport chegaram a dois tipos de conclusões: ou não revelaram efeitos positivos nem negativos dos programas; ou revelaram efeitos positivos quanto à sobrecarga experienciada pelo cuidador, sintomas de ansiedade e depressão e outros indicadores de saúde mental. Caso seja alocado ao grupo 2, recebendo um manual digital, sabe-se que, à exceção dos casos em que existe possibilidade do cuidador informal participar em iniciativas pontuais de programas de intervenção presencial, o manual digital replicará aquela que é na prática a intervenção máxima atualmente disponibilizada a estes cuidadores.

**Responsáveis pelo estudo e financiamento**: Este estudo encontra-se a ser desenvolvido no CINTESIS - Centro de Investigação em Tecnologias e Serviços de Saúde, sendo da responsabilidade da investigadora principal Soraia Teles (Psicóloga e investigadora deste centro) e da equipa de supervisão científica Constança Paul (Psicóloga, Professora Catedrática no ICBAS) e Ana Margarida Ferreira (Investigadora, especialista em Ciências da Computação). O estudo conta ainda com a parceria da Associação Alzheimer Portugal. O estudo será conduzido com recurso a financiamento próprio da investigadora principal e da instituição à qual se encontra afiliada. A realização deste estudo mereceu parecer favorável da Comissão de Ética para a Saúde do Centro Hospitalar de S. João/FMUP.

Para esclarecer qualquer dúvida ou fazer qualquer comentário poderá contactar a investigadora principal do estudo através do contacto móvel 916876843 ou do email teles.s.soraia@gmail.com.

Com os melhores cumprimentos e sempre disponível para esclarecimentos adicionais,

A investigadora principal,

______________________________________________________________

Soraia Teles, Psicóloga, investigadora.

CONSENTIMENTO INFORMADO, ESCLARECIDO E LIVRE

PARA PARTICIPAÇÃO EM INVESTIGAÇÃO

Considerando a “Declaração de Helsínquia” da Associação Médica

Mundial (Helsínquia 1964; Tóquio 1975; Veneza 1983; Hong Kong 1989;

Somerset West 1996, Edimburgo 2000, Seoul 2008, Fortaleza 2013)

**Designação do Estudo**

Apoio e formação *online* para cuidadores informais de pessoas que vivem com demência: Estudo de eficácia do programa iSupport.

*Confirmo que expliquei ao participante, de forma adequada e compreensível, a investigação referida, os benefícios, os riscos e possíveis complicações associadas à sua realização, em concordância com a informação escrita ao participante.*

| Informação escrita em anexo: | | |  | Não | x | Sim (Nº de páginas 3 | | | | ) |
| --- | --- | --- | --- | --- | --- | --- | --- | --- | --- | --- |
|  |  | |  |  |  |  |  |  |  |  |
| **O Investigador responsável** | | | |  |  |  |  |  |  |  |
| Nome: | | | | Soraia Teles de Sousa | | |  |  |  |  |
|  |  |  |  |  |  |  |  |  |  |  |
|  |  |  |  |  |  |  |  |  |  | (assinatura digital) |
| **Identificação do participante** | | | |  |  |  |  |  |  |  |
| Nome |  | | |  |  |  |  |  |  |  |
| BI/ CC nº | | | |  |  |  |  |  |  |  |
|  |  |  |  |  |  |  |  |  |  |  |

**Participante**

- Compreendi a explicação que me foi facultada acerca do estudo que se tenciona realizar: os objetivos, os métodos, os benefícios previstos, os riscos potenciais e o eventual desconforto.
- Solicitei todas as informações de que necessitei, sabendo que o esclarecimento é fundamental para uma boa decisão.
- Fui informado da possibilidade de livremente recusar ou abandonar a todo o tempo a participação no estudo, sem que isso possa ter como efeito qualquer prejuízo na assistência que é prestada.
- Declaro não ter sido incluído em nenhum outro projeto de investigação nos últimos três meses.

*POR FAVOR SELECIONE A OPÇÃO QUE SE ADEQUE À SUA DECISÃO DE PARTICIPAR/NÃO PARTICIPAR NESTE ESTUDO.*

🞎 ***Não concordo*** *com a participação neste estudo de acordo com os esclarecimentos que me foram prestados na informação ao participante, a qual posso guardar em formato digital e/ou imprimir.*

🞎 ***Concordo*** *com a participação neste estudo de acordo com os esclarecimentos que me foram prestados na informação ao participante, a qual posso guardar em formato digital e/ou imprimir.*

| Data: | | / | | | / | |  |  |  |  |
| --- | --- | --- | --- | --- | --- | --- | --- | --- | --- | --- |
|  |  |  |  |  |  |  |  |  |  |  |
|  |  |  |  |  |  |  |  |  |  |  |
